# Histological Severity Risk Factors Identification in Juvenile-Onset Recurrent Respiratory Papillomatosis: How Immunohistochemistry and AI Algorithms Can Help?

**DOI:** 10.3389/fonc.2021.596499

**Published:** 2021-03-08

**Authors:** Charles Lépine, Paul Klein, Thibault Voron, Marion Mandavit, Dominique Berrebi, Sophie Outh-Gauer, Hélène Péré, Louis Tournier, Franck Pagès, Eric Tartour, Thomas Le Meur, Sylvain Berlemont, Natacha Teissier, Mathilde Carlevan, Nicolas Leboulanger, Louise Galmiche, Cécile Badoual

**Affiliations:** ^1^INSERM-U970, PARCC, Université de Paris, Paris, France; ^2^Department of Pathology, Hôpital Européen Georges-Pompidou, APHP, Paris, France; ^3^KeenEye, Paris, France; ^4^Department of Pathology, Hôpital Robert Debré, APHP, Paris, France; ^5^Department of Virology, Hôpital Européen Georges-Pompidou, APHP, Paris, France; ^6^Department of Immunology, Hôpital Européen Georges-Pompidou, APHP, Paris, France; ^7^Department of Pediatric ENT Surgery, Hôpital Robert Debré, APHP, Paris, France; ^8^Department of Pediatric ENT Surgery, Hôpital Necker-Enfants Malades, APHP, Paris, France; ^9^Department of Pathology, Hôpital Necker-Enfants Malades, APHP, Paris, France

**Keywords:** juvenile onset recurrent respiratory papillomatosis, machine learning, deep learning, p53, p63, HPV, immunohistochemistry

## Abstract

Juvenile-onset recurrent respiratory papillomatosis (JoRRP) is a condition characterized by the repeated growth of benign exophytic papilloma in the respiratory tract. The course of the disease remains unpredictable: some children experience minor symptoms, while others require multiple interventions due to florid growth. Our study aimed to identify histologic severity risk factors in patients with JoRRP. Forty-eight children from two French pediatric centers were included retrospectively. Criteria for a severe disease were: annual rate of surgical endoscopy ≥ 5, spread to the lung, carcinomatous transformation or death. We conducted a multi-stage study with image analysis. First, with Hematoxylin and eosin (HE) digital slides of papilloma, we searched for morphological patterns associated with a severe JoRRP using a deep-learning algorithm. Then, immunohistochemistry with antibody against p53 and p63 was performed on sections of FFPE samples of laryngeal papilloma obtained between 2008 and 2018. Immunostainings were quantified according to the staining intensity through two automated workflows: one using machine learning, the other using deep learning. Twenty-four patients had severe disease. For the HE analysis, no significative results were obtained with cross-validation. For immunostaining with anti-p63 antibody, we found similar results between the two image analysis methods. Using machine learning, we found 23.98% of stained nuclei for medium intensity for mild JoRRP vs. 36.1% for severe JoRRP (*p* = 0.041); and for medium and strong intensity together, 24.14% for mild JoRRP vs. 36.9% for severe JoRRP (*p* = 0.048). Using deep learning, we found 58.32% for mild JoRRP vs. 67.45% for severe JoRRP (*p* = 0.045) for medium and strong intensity together. Regarding p53, we did not find any significant difference in the number of nuclei stained between the two groups of patients. In conclusion, we highlighted that immunochemistry with the anti-p63 antibody is a potential biomarker to predict the severity of the JoRRP.

## Introduction

Recurrent respiratory papillomatosis (RRP) is characterized by the repeated growth of benign exophytic papilloma in the respiratory tract (1, 2), primarily in the larynx (1). The age distribution of RRP in Europe is trimodal with a peak in children at a median age of 7 years and two other peaks in adults at a median age of 35 and 64 years old (3). This rare condition is referred to as Juvenile-onset Recurrent Respiratory Papillomatosis (JoRRP) when it occurs in children. Epidemiologic data vary depending on the country. In France, there are no available data. In Denmark, between 1969 and 1984, the incidence was 3.6 cases per year per 100,000 children (4). In Canada, based on a national database, the incidence and prevalence from 1994 to 2007 were respectively 0.24 per 100 000 children and 1.11 per children, median age at diagnosis was 4.4 years with a sex ratio close to 1:1 (5). In the United States, data are similar (6), however incidence and prevalence seem correlated to the socioeconomic status (7). JoRRP is caused by an HPV infection, mostly by genotypes 6 and 11 (8). These epidemiological data may change in countries with a strong HPV vaccination policy: an Australian study shows a decrease in the incidence of RRP in children under 14 years of age after the introduction of the national HPV vaccination program in 2007. The incidence decreased from 0.16 cases per 100,000 children in 2012 to 0.02 cases per 100,000 in 2016 (*p* = 0.034) (9). Three modes of transmission are suggested: vertical transmission at birth [HPV type concordance between mother and newborn in different studies are however contradictory (10–12)], vertical transmission in utero (13) and horizontal transmission via the child's environment (10). Whatever the transmission mode, several studies have demonstrated that maternal condyloma at the time of delivery was a major risk factor of developing JoRRP (14, 15). While the prevalence of HPV 6 and 11 infection in pregnant women is around 2%, the prevalence of JoRRP is surprisingly low. Thus, HPV infection alone does not explain the development of the disease and strong arguments suggest that JoRRP is tied to immunity defects and genetic susceptibilities. Patients with RRP are associated with HLA DRB1^*^0102/0301, DQB1^*^0201/0202 (16, 17) and present a lack of KIR genes 3DS1 et 2DS1 (18). Moreover, their immune response presents a Th2 polarization (19) which is not suitable for viral infection control. The management of this disease is challenging because its evolution remains unpredictable: some children experience minor symptoms with spontaneous remission, while others undergo multiple interventions due to florid growth. For the most severe cases, JoRRP may lead to airway compromise, and malignant transformation to carcinoma can occur, although it is extremely rare [most often over pulmonary spread (20, 21)]. The standard treatment of JoRRP is a surgical excision (SE) with cold instruments or microdebriders. Multiple endolaryngeal procedures can lead to glottis synechia and irreversible damage to the vocal cords as well as impaired social life (22). To improve the surgical outcome and extend symptom-free periods, numerous adjuvant treatments have been tried: interferon α (23), celecoxib (24), bevacizumab (25), cidofovir (26, 27), PD-1/PD-L1 immunotherapy (28, 29), and the quadrivalent HPV vaccine (30). At the time of writing, none of these treatments have been recommended for routine use by the International Pediatric Otolaryngology Group (31). The most promising ones are the quadrivalent HPV vaccine, bevacizumab and PD-1/PD-L1 immunotherapies which appear to decrease relapses (28, 29, 32, 33).

In light of the multiplication of neo-adjuvant treatments and the impossibility to predict the evolution of the disease, we have sought to identify severity risk factors in order to improve the handling of these children. Although many studies have focused on clinical severity risk factors, the only one identified to date is the early age of onset of the disease (34, 35). To our knowledge only one article investigated in JoRRP histological criteria related to disease severity (such as the presence of mitosis above the basal cell layer) but without significant results (36). Several studies have looked for histological criteria with the help of immunohistochemistry. Ahn et al. (37) studied the density of cells expressing CD8, CD4, FoxP3, PD-1, or PD-L1 in papilloma samples in a cohort of 39 patients. Only CD8+ cells density was inversely correlated with disease severity (*p* = 0.01). Another study on papilloma samples involving 12 patients found a trend between a greater number of cells marked by the anti-p53 antibody and greater disease activity (defined by more than 3 SE per year); however this association was not statistically significant (*p* = 0.1) (38). As a reminder, TP53 is a tumor suppressor gene, so its loss of function leads to tumor development. The p53 protein acts as a transcription factor regulating the expression of a large number of genes involved in the cell cycle, apoptosis, cell differentiation, DNA repair, cell metabolism, migration and angiogenesis (39). p53 immunohistochemistry is used as a prognostic factor (40, 41). It is also used to distinguish dysplastic epithelium (overexpressing p53) from epithelium with reactive changes (presenting a wild-type staining) (42). The p63 protein is a transcription factor belonging to the same family as the p53 protein. p63 protein appears to play an important role in the development of squamous epithelium (43). Given the scarcity of data in the literature on histological criteria associated with JoRRP severity, we decided to conduct this multi-stage study assisted by computerized image analysis. From Hematoxylin and eosin (HE) digital slides of papilloma, we first focused on morphological patterns associated with severe JoRRP. Finding morphological predictive patterns on HE slides could help optimize patient management. To our knowledge, no study has yet been able to find such criteria; and no computerized analysis was performed to determine such morphological criteria in this pathology. Thus, we extended our queries about potential morphological discriminative patterns using artificial intelligence. Indeed, artificial intelligence has an increasing impact on digital pathology as a help for decision-making that could usher in an acceleration of clinical workflows: several models showed a capability to recapitulate patterns that experts had already recognized (44). Some previous works even succeeded in predicting gene mutation on HE slides using deep-learning algorithms (45). In parallel, we explored p53 and p63 expressions with immunohistochemistry as potential markers of JoRRP severity, and compared quantitative results with two automated workflows: one based on machine learning, a second one based on deep learning. Machine learning refers to mathematical models that are designed to learn from experience, in order to make predictions or decisions without being explicitly programmed to do so. A machine-learning algorithm might require extraction of intermediate handcrafted features, for example typical cell size, or staining intensity histogram for a given object. The algorithm would base its prediction on these selected features. Deep learning is a subtype of machine learning that goes even beyond: the model learns and builds by itself relevant features to make a final prediction, making it more generalizable and unbiased in the way features are extracted. Our step toward a deep-learning-based approach was supported by the overwhelming majority of state-of-the-art architectures that now rely on deep learning in every computer vision task. We relied on both approaches to strengthen our conclusion and ensure a high confidence in our final quantitative results.

## Materials and Methods

### Population

This retrospective study was approved by an ethical committee (notice number: CPP2019-02'-019a/2019-00352-55/19.02.05.67237) and by the “Commission Nationale Informatique et Libertés” (application number: 919150). Patients were selected from two pediatric University Hospital Centers (CHU) treating JoRRP: Necker-Enfants Malades Hospital and Robert Debré Hospital (both in Paris). Patients were selected by querying each hospital database via the laboratory management software Diamic for samples taken between 2008 and 2017 with the following diagnoses: juvenile papillomatosis, viral papilloma, squamous papilloma, and papillomatosis. The single most recent sample per patient was selected, thus allowing for the best possible slide quality to be obtained for immunohistochemistry. The inclusion criteria were:

- A positive HPV “low risk” DNA *in situ* hybridization test or a positive PCR targeting HPV 6 and/or 11.
- Recurrence after diagnosis.

Clinical data were collected retrospectively in March 2018, and gathered the following information: gender, exact age at diagnosis, dates of each SE performed in the two University Hospitals, number of SE, number of Cidofovir injections received, potential tracheostomy in relation to the disease, presence of surgical sequelae (defined as the appearance of synechia of the glottis or even stenosis), location of papilloma lesions, presence of lung involvement (proven by at least one chest CT scan), presence of a lesion at the last flexible endoscopy, notion of carcinomatous transformation, potential death related to the disease.

From the dates of the SE, an average interval in days between each SE was calculated. The number of SE per year was calculated by dividing the total number of SE by the number of years between the first and last SE.

### HPV Typing

When the HPV type was not already known, FFPE papilloma samples from the patient were sent to the Georges Pompidou European Hospital's Virology Department, where PCR were performed with the INNO-LiPA® kit from Innogenetics, targeting 28 HPV genotypes including 6 and 11.

### Immunohistochemistry and Staining

Immunohistochemistry was performed on sections of FFPE tissue samples of laryngeal papilloma with anti-p53 (Dako, DO-7 clone, 1/50 dilution) and anti-p63 antibodies (Roche, 4A4 clone, 1/50 dilution) carried out on a Leica™ Bond III® automat according to the protocols routinely used in the pathology department of the Necker-Enfants Malades hospital.

For each patient, we also collected an HE slide of the same laryngeal papilloma used for immunohistochemistry. Each HE slide contained at least one and up to six levels.

### Image Analysis

Each p53 and p63 immunohistochemistry was scanned with a Vectra Polaris® slide scanner from Akoya Biosciences™ with a magnification corresponding to a 10x objective. Each HE slide was scanned with a NanoZoomer® from Hamamatsu® with a magnification corresponding to a 40× objective.

### Prediction of Disease Severity Using Solely HE With a Deep Neural Network

We decided to apply a deep neural architecture to classify HE slides into mild or severe JoRRP, and potentially unveil what was learned by the model to highlight specific tissue regions that activated the decision.

**Figure 1:**
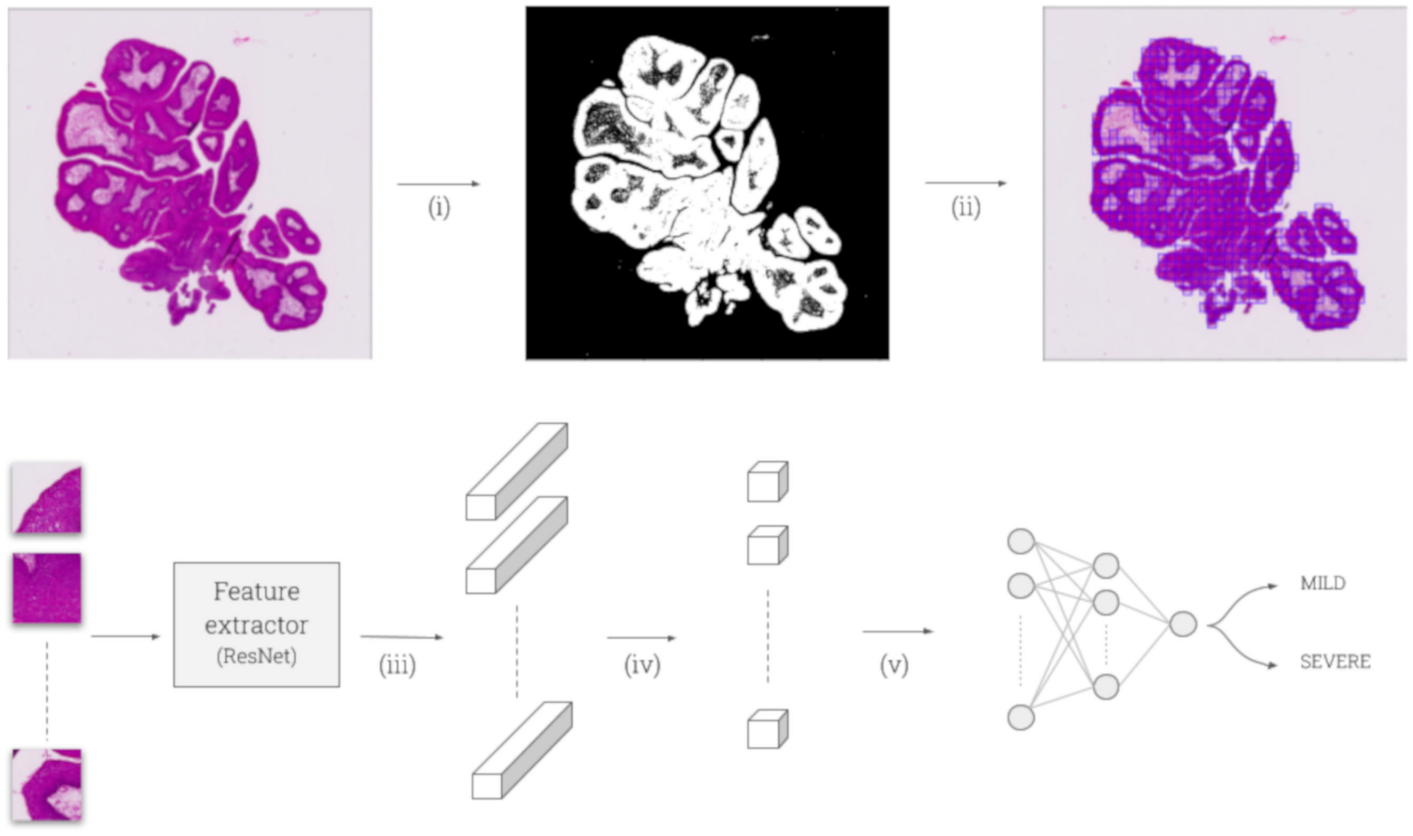
(i) WSI is thresholded with Otsu to separate foreground (tissue) from background. (ii) Selected foreground is cut out into tiles of size 224 × 224 pixels. (iii) Tiles are fed into a convolutional feature extractor—here ResNet-50, pretrained on ImageNet—leading to a 2,048 feature vector for each tile, after the 5th ResNet block and mean pooling. (iv) A 1-by-1 convolution is applied to get a single value per feature vector. (v) Tiles scores are sorted: *R* maximal scores and *R* minimal scores are selected to go through a final two-layer perceptron (200 and 100 hidden units) to make a final softmax prediction of the class: either “mild” or “severe.” We set *R* = 5 as suggested by CHOWDER.

**Figure 2:**
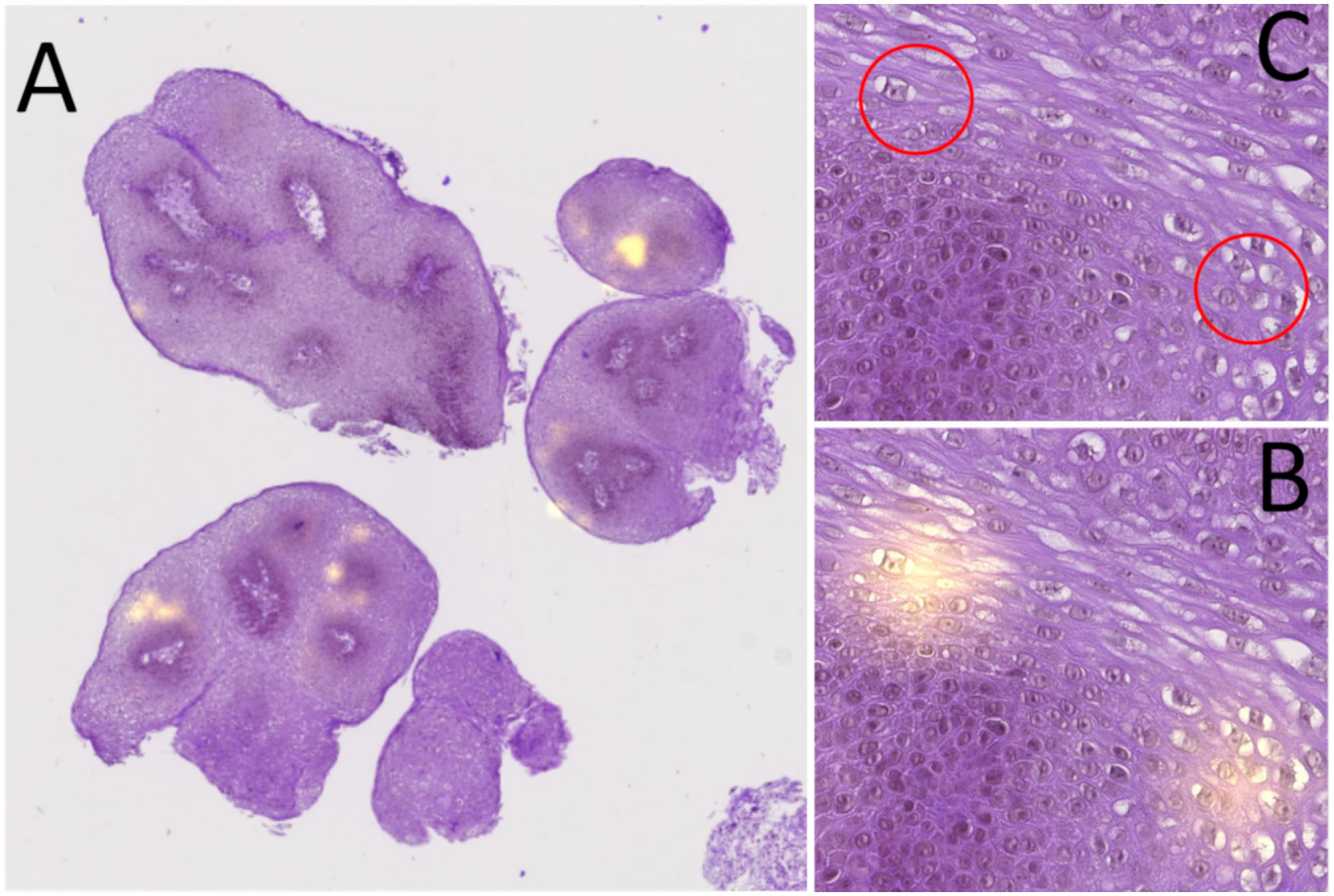
Heatmap analysis process: **(A)** global view of an HE slide with its heatmap; the yellow zones represent the areas that have impacted the classifier (hotspots). **(B)** View of two heatmap hotspots. **(C)** HE area corresponding to the two hotspots, allowing to see the presence of viral cytopathogenic effect (red circle corresponding to the hotspots).

We designed a deep-learning architecture relying on CHOWDER (46), an end-to-end framework that extended WELDON (47) for Whole Slide Images (WSI) classification: the goal of such network is to classify WSI into classes of interest (mild and severe JoRRP). Due to the size of WSI (typically 100,000 × 100,000 pixels), it is not possible to pass an entire digitized slide as is through a neural network due to memory limitations. To overcome these, tissue regions are located with Otsu thresholding (48), and are then cut out into tiles (of size 224 × 224 pixels). A score is attributed to each of these tiles by a convolutional neural network, then aggregated through a fully connected network to make a final decision. The full architecture is described in Figure 1. We also worked on unveiling which specific tiles activated the final decision. For each evaluation slide, we extracted the tiles to which the model was paying the most attention and highlighted them via heatmaps, as shown in Figure 2.

We validated our implementation by collecting 1,580 non-Small Lung Carcinoma (NSLC) H&E slides, made publicly available by The Cancer Genome Atlas (TCGA). The details of the validation steps of our model and the heatmaps are described in the Supplementary Data.

Using such a large cohort allowed us to validate our implementation with an overall AUC of 0.966 to predict cancer types, thus reaching high classification performance on a task already managed by pathologists. However, our JoRRP cohort was small by the standards of such WSI classification task in machine-learning community (*n* = 48), so we tried different approaches described below, to synthetically increase the dataset size and to regularize the model. We used a four-fold cross-validation procedure in all our experiments, to confirm that our method could be generalized over an independent dataset, and flag problems such as overfitting or selection bias. Thus, for each experiment, the JoRRP cohort was splitted into four subsets, and four models were trained: each one was trained with three subsets and evaluated on the remaining one. The performance is then reported as the average of the four models performances. To address data scarcity, we tested different ways to augment and regularize our training set: basic data augmentation on tiles (flip, rotations), increasing training set size by considering different neighboring slices as independent cases, using bags dropout (49) by randomly sending a subset of the input tiles in the network (90, 80, and 70% were tested out), using a pretrained ResNet-50 feature extractor (on ImageNet and on TCGA-lung). Additionally, we experimented with different magnification levels for tiling (20X, 10X, and 5X), to ensure we scanned all potentially relevant morphological structures.

### Machine-Learning Approach for p53 and p63 Immunohistochemistry

**Figure 3:**
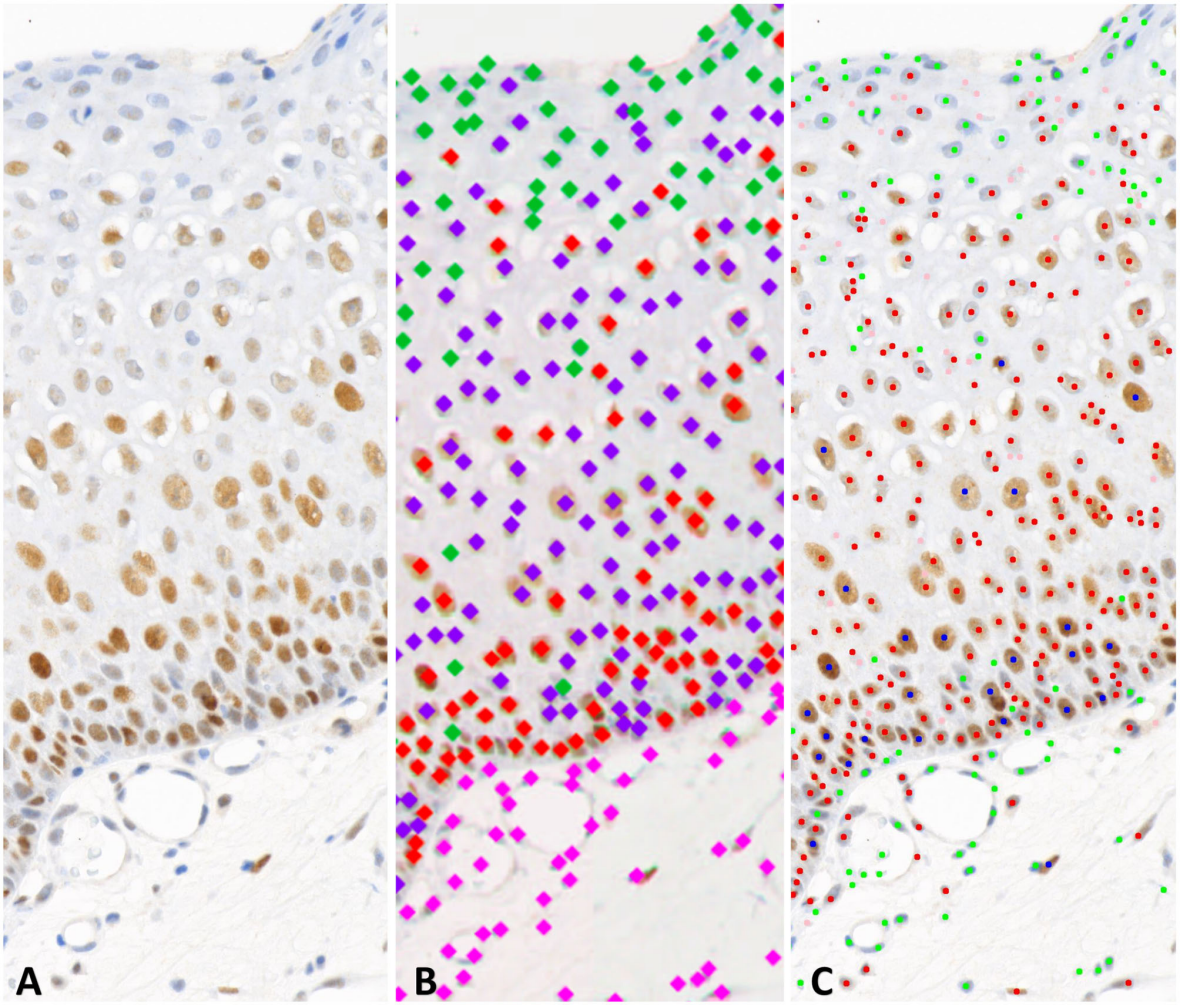
Examples of the machine-learning and deep-learning phenotyping. **(A)** area of an ROI from a p53 slide. **(B)** Deep-learning approach, labeling of the colors: pink, stroma; green, unstained; purple, low intensity staining; red, medium intensity staining. **(C)** Machine-learning approach, labeling of the colors: pink, irrelevant; green, unstained; red, low intensity staining; blue, medium intensity staining.

p53 and p63 quantitative analysis was performed with the Inform® 2.3 software from Akoya Biosciences™, which enables users to fine-tune built-in quantification algorithms. The analysis is a two-stage procedure: nuclei segmentation and nuclei phenotyping. Nuclei segmentation was performed by the software based on the DAB algorithm provided by the manufacturer. Then, for nuclei phenotyping, the model, which was based on multinomial logistic regression, needed to be trained to perform phenotyping. We thus selected 13 regions of interest from virtual immunohistochemistry slides of p53 (9 ROI) and p63 (4 ROI) antibodies, and had them annotated by a pathologist. Each region of interest came from a different patient, to foster staining expression and morphological heterogeneity within the training set. We gathered a training set of 500 annotated nuclei in these fields, with five labels as described in Figure 3 [weak (1+), medium (2+) and strong staining (3+), unstained and irrelevant for non-nuclei objects]. We manually labeled nuclei until the automatized recognition by the Inform® software was concordant with visual count on the training set. Once trained, we selected at least 8 regions per p53 and p63 virtual slide to run a full quantitative analysis. The size of a region of interest was 0.47 mm × 0.35 mm. Fields of interest were selected to contain only the entire surface of the papilloma epithelium with as little connective tissue as possible. They were then analyzed by the Inform® software trained algorithm and each region of interest analyzed was visually verified. Viray et al. (50) found high accuracy between the software results and manual analysis by pathologists, yet we quantitatively assessed the algorithm performance by comparing its predictions to a pathologist annotations. We randomly selected six regions of interest from two different patients, three ROI from p53 staining and three ROI from p63 staining, containing approximately a total of 4,000 nuclei. Results show a global positive predictive value of 0.83 and a global sensitivity of 0.95. In details, positive predictive value/sensitivity results per class are: unstained (0.92/0.95), weak staining (0.87/0.95), medium staining (0.94/0.98), strong staining (0.97/0.87), and irrelevant (0.80/0.90). At the end, data of each ROI were extracted with R software.

### Deep-Learning Approach for p53 and p63 Immunohistochemistry

For this approach, we selected a Faster R-CNN architecture (51) to perform cell localization and classification. This is a two-stage architecture that first tells the model where to look (with the Region Proposal Network), and then classifies the proposed objects among classes of interest. The model was trained on 10 regions of interest of size 0.512 mm × 0.512 mm, coming from five different slides (three p63 and two p53 slides). Each region was fully annotated by a pathologist with point annotations for each nuclei. Five classes were predefined: stroma, unstained (0), weakly stained (1+), moderately stained (2+), and strongly stained (3+). We chose to add a dedicated class for stroma (although this is not taken into account in staining level expression) to enforce the network to learn the distinction between stroma cells and unmarked epithelial cells despite their staining intensity similarities. By adding an extra class for stroma cells, we regularized the network and fostered morphological context learning to distinguish epithelium from stroma. The model was trained during 10,000 iterations with a weighted cross-entropy (weights equal to the inverse of the class frequency in the training set), a learning rate of 10–3, and Adam optimizer (52). As for the machine learning analysis, we randomly selected 6 regions of interest (size of 0.256 mm × 0.256 mm) from the same two patients, containing approximately a total of 3,000 nuclei. We reported a global positive predictive value of 0.90 and a global sensitivity of 0.91. In details, positive predictive value/sensitivity results per class are: unstained (0.98/0.83), weak staining (0.84/0.88), medium staining (0.92/0.98), strong staining (0.97/0.92), and stroma (0.84/0.91).

### Statistical Analysis

Statistical analyses were carried out using R software. For p53 and p63 immunohistochemistry, we calculated for each patient a percentage of nuclei stained by level of intensity from raw data, by dividing the number of nuclei in each category by the total number of nuclei, on all regions of interest. For the deep-learning approach, the nuclei in the stroma were not taken into account. Qualitative variables were analyzed with a Chi2 or Fisher test depending on sample size. Univariate analyses with quantitative data were performed using a non-parametric Mann–Whitney test. Finally, all tests were bilateral and a *p* < 0.05 was considered significant.

### Outcome

Patients were classified into two groups: severe and mild. Severity was defined by at least one of the following criteria: a number of SE per year ≥5, death related to disease, pulmonary location of JoRRP proven at least by a chest CT scan, carcinomatous transformation of an JoRRP localization. One of the aims of this work was to identify histological criteria associated with a severe JoRRP. As there are, to our knowledge, no existing morphological JoRRP severity criteria, we tried a hypothesis-agnostic approach by using a deep-learning algorithm to classify patients in each of the two groups according to the HE alone. If validated, such algorithm could be used to extract tissue areas on which the algorithm particularly relied to make its decision, thus potentially highlighting discriminating histological criteria. Given the small size of our dataset, hence limiting the potential of such algorithm, we also planned to stain slides with anti-p53 and anti-p63 antibodies. We compared the percentage of nuclei stained by these two antibodies between the two groups.

## Results

### Population

Forty-eight children were included, 22 boys and 26 girls. The average age at diagnosis was 3.8 years with a median age of 2 (age range: 0.5–13 years). Twenty-seven percent of patients had HPV 11 infection, 65% had HPV 6 infection, and 6% had co-infection with HPV 6 and 11. It was not possible to perform HPV typing in one patient due to sample depletion. All patients had glottic involvement. 73% of patients had supraglottic tumors, 68.7% had subglottic ones and 25% and 8% had respectively tracheal and pulmonary involvement. Patients had a median rate of 4.8 SE per year. Regarding adjuvant treatment, 73% of patients received at least one injection of Cidofovir. Patients received an average of 7.1 injections of Cidofovir with a median of 3.5 injections. Six patients (12.5%) received Cidofovir during an SE prior to the study specimen. The delay between the first and last SE was on average 3.6 years and the median was 2 years. Moreover, 71% of patients had a lesion at the last check-up. Additionally, a young patient in our cohort died at the age of 18 from the malignant transformation of a pulmonary localization of her JoRRP into bronchopulmonary squamous cell carcinoma. Her JoRRP progressed for 17 years: 132 SE were performed, with a mean interval between each endoscopy of 47 days. She also received 67 injections of Cidofovir. According to our severity criteria, 24 patients had a severe disease and 24 had a mild disease. Characteristics of the two populations are summarized in Table 1. The two populations were comparable: there were no statistically significant differences in the gender of the patients, the type of HPV, the age at diagnosis, the total number of SE, the number of SE in the first year, the total number of injections of Cidofovir, or post-surgical morbidity. Patients with severe disease had a significantly shorter mean interval between each SE compared with patients with mild disease (median 51 days vs. 213 days, *p* < 0.0001). Patients with severe JoRRP had a shorter delay between first and last SE (1.0 year vs. 2.7 years, *p* = 0.001); and had significantly more tracheostomies than patients with mild JoRRP (*p* = 0.048).

**Table 1:** Clinical characteristics of patients with mild and severe JoRRP.

		**Mild disease: 24 (%)**	**Severe disease: 24 (%)**	***p***
Gender	Boys	11 (46%)	11 (46%)	1
	Girls	13 (54%)	13 (54%)	1
HPV type^*^	HPV6 and 11	3 (12%)	0 (0%)	0.234
	HPV11	4 (17%)	9 (38%)	0.194
	HPV6	17 (71%)	14 (58%)	0.546
Tracheostomy		1 (4%)	7 (29%)	**0.048**
Sub-glottic involvement		14 (58%)	19 (79%)	0.119
Tracheal involvement		5 (21%)	7 (29%)	0.505
Postoperative morbidity		5 (21%)	5 (21%)	1
Lesion at last check-up		15 (63%)	19 (79%)	0.204
Pulmonary involvement		0	4 (17%)	
Death		0	1 (4%)	
Malignant transformation		0	1 (4%)	
Median age at diagnosis (year)		3	2	0.180
Median time between 1st and last SE (years)		2.7	1	**0.001**
Median total number of SE		8	9.5	0.193
Median number of SE first year after diagnosis		3.5	5	0.054
Median average interval between each SE (days)		213	51	**<0.0001**
Median total number of Cidofovir injections		3	5	0.311

* *One patient could not have HPV typing due to sample depletion*.

*Bold value indicate statistically significant (<0.05)*.

### Prediction of Disease Severity Using Solely HE With a Deep Neural Network

We tested different approaches (as described in our “Methods” section) to face data scarcity, which is an obstacle for such multiple instance learning tasks. Given the small evaluation set size for a given training (corresponding to 11–12 slides), running a cross-validation was compulsory to properly validate a method. Here, we systematically carried out a four-fold cross-validation. If one configuration sometimes gave good results on specific sets (we reached 0.83 AUC on a set with a single slice per patient, all tiles being used at each training iteration), we never reached significant results on cross-validation (mean AUC of 0.57 with a non-statistically significant *p*-value). Beyond evaluation metrics, we strove to understand whether the algorithm took into account histological criteria visible to a pathologist. To find potential histological criteria that would allow mild/severe stratification solely with HE slides, we randomly compared five heatmaps of patients with severe JoRRP with five heatmaps of patients with mild disease that had been accurately classified by the model. For each heatmap, we noted the different locations of the hotspots (in the three thirds of the epithelium and in the conjunctivo-vascular axis). We also collected the presence of visible histological signs in the hotspot area (presence of lymphocytes, neutrophil polynuclear cells, viral cytopathogenic effect, prominent nucleoli, nuclear hyperchromatism, and mitosis). The results are summarized in Supplementary Table 1. Briefly, according to the heatmaps analyzed, there was an average of 11 hotspots per patient. There is a slightly different distribution of hotspots depending on the severity of the disease, with more hotspots in the basal third and in the stroma for patients with severe disease and more hotspots in the middle third for patients with mild disease. We found 19 out of 27 hotspots with histological criteria. Some features are only found for patients with a mild JoRRP, such as a prominent nucleoli and mitosis. Neutrophils are only found for patients with a severe JoRRP.

### Image Analysis of p53 and p63 Immunohistochemistry

Given the small size of our dataset limiting the outcome of a WSI classification task, we also planned to stain slides with anti-p53 and anti-p63 antibodies. An example of the nuclei phenotyping results with each approach is shown in Figure 3.

#### Machine-Learning Approach

Concerning the machine-learning approach, results are summarized in Table 2. Patients with severe disease had statistically significant higher numbers of stained nuclei with anti-p53 antibody for strong intensity compared with patients with mild disease (0.14 vs. 0.08, *p* = 0.015). There was no significant difference for the other intensity groups. With the p63 antibody, patients with severe disease had statistically significant higher numbers of stained nuclei compared with patients with mild disease for medium intensity (36.1 vs. 23.98%, *p* = 0.041) and medium and strong intensity together (36.9 vs. 24.14%, *p* = 0.048).

**Table 2:** Comparison of the percentage of nuclei stained by antibody against p53 and p63 between patients with mild and severe JoRRP with the machine-learning approach.

	**Staining intensity**	**Mild disease (24)**	**Severe disease (24)**	***p***
% of nuclei stained by p53 antibody (median)	+	61.08	62.68	0.564
	++	3.2	4.67	0.073
	+++	0.08	0.14	**0.015**
	All of the 3	65.38	69.46	0.266
	++ and +++	3.36	4.91	0.063
% of nuclei stained by p63 antibody (median)	+	55.7	49.56	0.108
	++	23.98	36.1	**0.041**
	+++	0.14	0.74	0.122
	All of the 3	82.02	86.07	0.055
	++ and +++	24.14	36.9	**0.048**

*Bold value indicate statistically significant (<0.05)*.

#### Deep-Learning Approach

Concerning the deep-learning approach, results are summarized in Table 3. With the p63 antibody, patients with severe disease had statistically significant higher numbers of stained nuclei compared to patients with mild disease for the three intensities together (87.55 vs. 84.64%, *p* = 0.023) and medium and strong intensity together (67.45 vs. 58.32%, *p* = 0.045). There was no significant difference between the two populations regarding the number of nuclei stained by the p53 antibody.

**Table 3:** Comparison of the percentage of nuclei stained by antibody against p53 and p63 between patients with mild and severe JoRRP with the deep-learning approach.

	**Staining intensity**	**Mild disease (24)**	**Severe disease (24)**	**p**
% of nuclei stained by p53 antibody (median)	+	49.51	47.97	0.951
	++	14.42	16.51	0.483
	+++	0.08	0.18	0.085
	All of the 3	64.93	71.82	0.303
	++ and +++	14.59	16.94	0.483
% of nuclei stained by p63 antibody (median)	+	25.4	19.8	0.303
	++	53.85	57.65	0.201
	+++	0.84	3.94	0.066
	All of the 3	84.64	87.55	**0.023**
	++ and +++	58.32	67.45	**0.045**

*Bold value indicate statistically significant (<0.05)*.

## Discussion

### Population

Juvenile recurrent respiratory papillomatosis is a rare disease and studies often involve small cohorts, which severely limits their scope. In order to improve the management of these patients, it is necessary to carry out studies to find new severity risk factors. To our knowledge, our cohort of JoRRP is the largest ever studied in Europe. National databases in the U.S. and Canada have been established, covering 603 and 243 children with JoRRP (5, 53); our population has characteristics comparable to these two cohorts. We found a median rate of SE per year of 4.8 comparable to the U.S. cohort's, which was of 4.3, higher than the Canadian one of 1.5. Our median age at diagnosis was slightly lower, 2 years old vs. 3 years old in the U.S. cohort and 4 years old in the Canadian one. These data are also similar with a more recent publication on an international cohort of juvenile and adult RRP (35). Interestingly, the percentage of patients treated with Cidofovir was much higher in our cohort than in the Canadian cohort (respectively 73 vs. 4.7%). The differences in terms of Cidofovir treatment could be explained by variability in local practices. Regarding the distribution of HPV types, our data are comparable to the literature. We found a low proportion of co-infection with HPV6 and 11 (6%) and a predominance of HPV6 (65%), as described elsewhere (54, 55). One of the main difficulties in our study was to define disease severity. Currently, no consensual definition exists in the literature. Some authors use composite scores incorporating criteria for disease localization, such as the Derkay–Wiatrak score, and intervention-related criteria, such as the number of SE per year (5, 35, 56). Others use only intervention-related criteria. A total number of SE greater than or equal to 10 or a number of SE/year > 3 or 4 is frequently found as a criterion of severity (34). We were unable to use Derkay–Wiatrak score as one of the two centers involved was not used to performing it systematically. We chose the criteria mainly representing the symptomatology of these two groups of patients. Our cut-off value for the number of SE per year seems relevant for our cohort, since patients classified as severe presented more severe items of disease activity than patients classified as mild. Thus, the median mean interval between each endoscopy was 51 days for severe JoRRP and 213 days for mild JoRRP (*p* < 0.001). Additionally, patients with severe disease had statistically significantly more tracheostomies than those with mild disease (*p* = 0.048). It should be noted that as 71% of patients had a lesion at the last check-up it may be possible that the number of SE/year would have changed until remission.

### Prediction of Disease Severity Using Solely HE With a Deep Neural Network

The principal aim of this study was to identify histological criteria associated with disease severity. We first aimed to determine whether we could predict JoRRP severity solely relying on HE slides. The difficulty was twofold: the cohort was small for WSI classification tasks with respect to machine learning community standards, and this was a discovery task, meaning that there are no known predictive morphological discriminative patterns that distinguish severe from mild JoRRP for pathologists. Despite our efforts to address data scarcity, we did not find a configuration capable of performing well on all cross-validation sets. We concluded that our dataset did not make it possible to extract from HE slides the information relevant to predict JoRRP severity with our multiple instance learning approach. It shows that such architecture was not able to extract extra information as for a pathologist, at least on such small dataset. We acknowledge that it does not imply that no such morphological pattern in HE could be useful to predict JoRRP severity; yet, we think that highlighting what did not work is still an informative milestone for the community to design future projects. A larger transnational cohort would facilitate research and statistically strengthen the approach, given the difficulty of such discovery tasks. The classification model for JoRRP was not sufficiently effective to allow complete heatmaps analysis. However, it is very easy for a pathologist to analyze the areas used by the algorithm to classify a case. This may prove to be time-saving for the analysis of a cohort and helpful in identifying histological items potentially associated with the severity of the disease. Indeed, by simply exploring 5 cases, we found a slightly different distribution of the hotspots on the slides between the two groups and some differences in histological criteria found below the hotspots between mild and severe JoRRP. Even though it was not possible to draw conclusions from these data, this kind of analysis with secondary morphological analysis of area of interest seems promising for pathologists.

### Image Analysis of p53 and p63 Immunohistochemistry

Considering the lack of significant results on HE, we also explored p63 and p53 immunostainings. Based on Rabah et al. (38) results, we set out to explore the expression of p53 in these tumors, and by extension, of p63. We decided to compare percentages of stained nuclei rather than density of labeled cells because machine-learning analysis tends to segment large nuclei in half, artificially increasing the number of cells in the ROI. The contribution of automated image analysis in this study considerably helped us save time and strengthened the robustness of such quantitative task. For p53, we did not find any difference in number of nuclei stained between the two groups of patients, except for the machine-learning approach concerning strong intensity. However, there is little difference between the percentage of stained nuclei of the two groups (0.08% for mild JoRRP vs. 0.14% for severe JoRRP) and it is questionable whether this discrepancy with the deep-learning approach is related to the fact that some stromal cells were taken into account in the analysis with machine learning (as exposed in Figure 3). The inability to detect these stromal areas in the machine-learning analysis and to exclude them may induce a bias in the counting of stained nuclei. This is why we opted for two distinct approaches for image analysis, deep learning allowing a finer analysis by taking into account the tumor cells exclusively, not the stromal cells. Indeed, our analysis with Inform® software did not allow distinction between these two types of cells. Moreover, these results are consistent with other studies that have looked at the expression of p53 in RRP. Stern et al. (57) found an higher percentage of p53 positive cells in patient that underwent malignant transformation than in tumors with benign course (68.3 vs. 14.2%, *p* < 0.05), however only 4 had malignant transformation over the 35 patients included and no correlation with other aggressiveness disease criteria was found. Perdana et al. (58) also reported no correlation between severity and the expression of p53. With the p63 antibody, the stromal cells are not stained but are counted as unstained cells by the algorithm. We found similar results between the two image analysis methods for anti-p63 antibody for medium and high intensities together, with a greater number of nuclei stained with these intensities in patients with severe disease. For each approach, we found around 10% differences in labeled cells between severe JoRRP and mild JoRRP (37 vs. 24% for machine learning and 67 vs. 58% for deep learning). These gross percentage differences between machine-learning and deep-learning approaches could be explained by the detection of stromal cells, which were detected as unstained nuclei with machine-learning approach, artificially biasing results. There may also have been a slight variability in the pathologist's annotation of the different classes for each approach, since his eyes were the only judge of the intensity of the staining. Nevertheless, the same pathologist made the annotations for both approaches, limiting variability. On the other hand, the deep-learning approach seemed more reliable since the model was trained to differentiate stromal from epithelial cells based on morphological context regardless of staining intensity. Obtention of similar results with the two image analysis methods strengthened the reliability of these results. Additionally, the positive predictive values and sensitivity of both models are very good. The fact that patients with severe disease had a higher percentage of cells labeled with p63 for medium and high intensities than patients with mild disease is a first step toward using p63 as a predictor of disease severity. A possible confounding factor in our study is the blend of patients treated or not treated with Cidofovir. Indeed, it is described in the literature that in HPV-induced cancer cell lines, Cidofovir causes an accumulation of p53 (59). However, these data concern high-grade HPV, and the low-risk HPV proteins involved here in JoRRP do not share the same properties. The E6 protein has a lower affinity for p53, which does not induce p53 degradation but retains an inhibitory activity to the p53 transcriptional activity necessary for viral genome replication (60). It is thus difficult to extrapolate the role of Cidofovir on the expression of p53 in JoRRP. We also analyzed our cohort in subgroups to take into account Cidofovir treatment, results are summarized in Supplementary Tables 2, 3. However, these results are difficult to interpret given that the groups are very disproportionate in size, as 73% of patients received at least one injection of Cidofovir. Even if Cidofovir has an impact on p53 expression in JoRRP, in our cohort the patient groups with a mild or a severe disease are well balanced with 29 and 25% of untreated patients respectively (7 patients out of 24 vs. 6 patients out of 24, *p* = 0.745). To confirm our results, a national prospective cohort with a larger number of patients will have to be set up with samples before and after injection of Cidofovir to study the impact of the latter on the expression of our markers.

## Conclusion

In conclusion, we highlighted that patients with a severe JoRRP presented a higher percentage of cells stained by the anti-p63 antibody for medium and strong intensities compared to patients with mild JoRRP. This was not found with the anti-p53 antibody. Use of a biomarker to predict an aggressive disease could allow to implement adjuvant treatment at the early stage of the disease. It could also be an opportunity to better inform patients and their parents of the potential course of the disease. We also presented an innovative approach in digital pathology, which consists in analyzing an area taken into account by a deep-learning algorithm for its predictions in an attempt to discover new histological criteria of severity in this disease. These analyses were possible thanks to close collaboration between pathologists and data scientists, and this should inspire us in the future development of our profession as pathologists. These data are a first step toward a better prediction of severe cases and better management tailored to the severity of JoRRP.

## Data Availability Statement

The raw data supporting the conclusions of this article will be made available by the authors, without undue reservation.

## Ethics Statement

The studies involving human participants were reviewed and approved by CPP2019-02'-019a/2019-00352-55/19.02.05.67237. Written informed consent from the participants' legal guardian/next of kin was not required to participate in this study in accordance with the national legislation and the institutional requirements.

## Author Contributions

Conceptualization: LG and CB; methodology: CB, CL, LG, SB, and PK; validation: CB, CL, LG, MM, and PK; formal analysis: CL, TV, and PK; investigation: CL, PK, and MM; resources: LG, DB, NT, NL, and FP; data curation: CL LT, and MC; writing — original draft preparation: CL and PK; writing — review and editing: LG, NT, CB, SO-G, ET, SB, TM, PK, and CL; visualization: CL; supervision: CB, LG, and SB; project administration: CB and CL; funding acquisition: CB, CL, LG, NL. All authors have read and agreed to the published version of the manuscript.

## Conflict of Interest

The authors declare that the research was conducted in the absence of any commercial or financial relationships that could be construed as a potential conflict of interest. The reviewer RS declared a past co-authorship with one of the authors NL to the handling editor.
