# Supplementary material for: Histological Severity Risk Factors Identification in Juvenile-Onset Recurrent Respiratory Papillomatosis: How Immunohistochemistry and AI Algorithms Can Help?
Source: Front Oncol. 2021 Mar 8;11:596499. doi: 10.3389/fonc.2021.596499 (PMC7982831; doi:10.3389/fonc.2021.596499)
Supplement: Supplementary file 1 [file Table_1.docx]

**Supplementary data**

**Validation of our implementation over a dataset of non-Small Lung Carcinoma (NSLC) H&E slides and heatmap analysis.**

Our model was trained to classify each sample into one of the 3 classes: normal, squamous carcinoma (LUSC) and adenocarcinoma (LUAD). In total, we gathered 480 normal, 576 LUAD and 527 LUSC HE slides. We used 80% of the dataset for training and validation purposes and kept 20% aside to evaluate the model performances once trained. This partitioning was stratified, meaning classes distribution were identical between training and tests sets. The model predicted the histological type with an accuracy of 0.883 on the 316 slides from the test set. We additionally reached an overall AUC of 0.966 (AUC-LUAD=0.944, AUC-LUSC=0.958, AUC-normal=0.996).

Regarding the heatmaps, allowing to highlight tiles to which the model was paying the most attention, we randomly selected 30 correctly classified WSI – 15 from each type of cancer. Using the heatmap with the hotspots as guidance, a pathologist reviewed each slide. He qualitatively assessed the cases and confirmed the model’s ability to correctly identify the relevant region that characterizes the specific lung cancer subtype: tumor cells characterized by a squamous appearance, similar to the one observed in epidermal cells for LUSC for example as shown in Supplementary Figure 1.


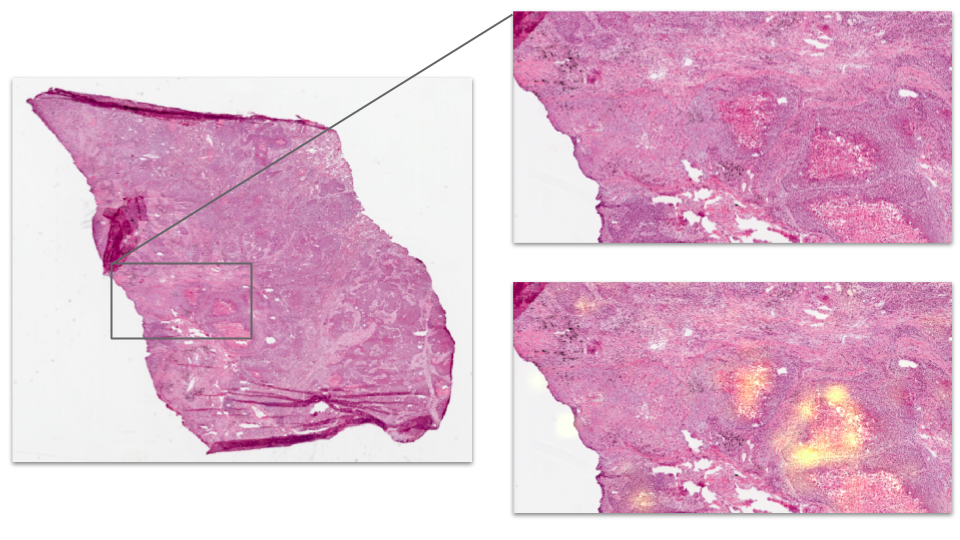


Supplementary Figure 1: On a LUSC slide (left image) accurately classified by the model, top contributing areas to make the prediction have been highlighted in yellow (bottom right image) for review. Above, the raw region, without hotspots.

|  | | Mild disease (n=5) | Severe disease (n=5) |
| --- | --- | --- | --- |
| Mean total number of hotspots (standard deviation) | | 11 (7) | 11 (7.6) |
| Mean number of hotspots according to the location (standard deviation) | basal third | 3 (1.6) | 5 (5) |
|  | middle third | 7 (4.3) | 3 (1.1) |
|  | superficial third | 3 (2.8) | 3 (0.9) |
|  | stroma | 0 | 3 (2.8) |
| Mean number of hotspots with a histological criteria (standard deviation) | viral cytopathogenic effect | 5 (5.5) | 3 (0.8) |
|  | nuclear hyperchromatism | 0 | 0 |
|  | Prominent nucleoli | 2 (0.5) | 0 |
|  | Mitosis | 1 | 0 |
|  | lymphocytes | 3 (0.5) | 3 (2.5) |
|  | neutrophils | 0 | 2 (1.4) |

Supplementary Table 1: location and histological characteristics of heatmaps

| **Supplementary Table 2**: Comparison of the percentage of nuclei stained by antibody against p53 and p63 between patient with mild and severe JoRRP with the machine-learning approach. | | | | | | | |
| --- | --- | --- | --- | --- | --- | --- | --- |
|  | | **Patient with adjuvant treatment** | | | **Patient without adjuvant treatment** | | |
|  | **Staining intensity** | **Mild disease (17)** | **Severe disease (18)** | **p** | **Mild disease (7)** | **Severe disease (6)** | **p** |
| **% of nuclei stained by p53 antibody (median)** | **+** | 57.41 | 57.92 | 0.807 | 62.74 | 59.68 | 0.445 |
|  | **++** | 2.83 | 3.85 | 0.318 | 3.42 | 7.72 | 0.014 |
|  | **+++** | 0.07 | 0.09 | 0.184 | 0.046 | 0.36 | 0.008 |
|  | **All of the 3** | 61.82 | 65.22 | 0.463 | 66.28 | 67.82 | 0.295 |
|  | **++ and +++** | 3.04 | 4.03 | 0.303 | 3.54 | 8.09 | 0.014 |
| **% of nuclei stained by p63 antibody (median)** | **+** | 58.27 | 51.01 | 0.143 | 48.94 | 37.37 | 0.295 |
|  | **++** | 22.36 | 34.06 | 0.195 | 31.33 | 43.42 | 0.035 |
|  | **+++** | 0.07 | 0.25 | 0.232 | 1.08 | 3.74 | 0.295 |
|  | **All of the 3** | 81.85 | 84.36 | 0.424 | 82.24 | 87.17 | 0.002 |
|  | **++ and +++** | 22.4 | 34.34 | 0.153 | 32.87 | 49.13 | 0.035 |
| **Supplementary Table 3**: Comparison of the percentage of nuclei stained by antibody against p53 and p63 between patient with mild and severe JoRRP with the deep-learning approach. | | | | | | | |
|  | | **Patient with adjuvant treatment** | | | **Patient without adjuvant treatment** | | |
|  | **Staining intensity** | **Mild disease (17)** | **Severe disease (18)** | **p** | **Mild disease (7)** | **Severe disease (6)** | **p** |
| **% of nuclei stained by p53 antibody (median)** | **+** | 49.49 | 46.31 | 0.708 | 52.30 | 50.77 | 0.836 |
|  | **++** | 14.49 | 15.22 | 0.961 | 12.39 | 19.08 | 0.234 |
|  | **+++** | 0.09 | 0.10 | 0.525 | 0.063 | 0.35 | 0.005 |
|  | **All of the 3** | 63.68 | 70.26 | 0.684 | 70.07 | 74.48 | 0.295 |
|  | **++ and +++** | 14.74 | 15.26 | 0.961 | 12.39 | 19.61 | 0.234 |
| **% of nuclei stained by p63 antibody (median)** | **+** | 26.16 | 20.09 | 0.483 | 19.74 | 13.04 | 0.366 |
|  | **++** | 51.66 | 57.65 | 0.232 | 56.73 | 58.53 | 0.731 |
|  | **+++** | 0.76 | 1.91 | 0.207 | 1.40 | 12.94 | 0.073 |
|  | **All of the 3** | 81.78 | 87.55 | 0.083 | 84.65 | 87.57 | 0.101 |
|  | **++ and +++** | 55.56 | 66.94 | 0.126 | 64.91 | 72.45 | 0.181 |
